# Dietary Ethanolamine Increases Hepatic Lipid Accumulation in Mice Fed a High-Fat Diet

**DOI:** 10.1016/j.tjnut.2025.101348

**Published:** 2026-01-08

**Authors:** Courtney M Holdaway, Amy Vo, Kelly-Ann Leonard, Randal Nelson, Aducio Thiesen, Yi Fan, Camila S Marcolla, Robin D Clugston, Benjamin P Willing, Rene L Jacobs

**Affiliations:** 1 Department of Agricultural, Food & Nutritional Science, University of Alberta, Edmonton, Canada; 2 Department of Physiology, University of Alberta, Edmonton, Canada; 3 Department of Laboratory Medicine, University of Alberta, Edmonton, Canada; 4 Agricultural and Agri-food Canada, Lacombe, Alberta, Canada

**Keywords:** ethanolamine, metabolic dysfunction-associated steatotic liver disease (MASLD), microbiome, phospholipids, phosphatidylethanolamine (PE)

## Abstract

**Background:**

Ethanolamine (Etn), a precursor of phosphatidylethanolamine (PE), may alter hepatic lipid homeostasis and gut health; its dietary effects remain undefined.

**Objective:**

The objective of this study was to determine the effects of dietary Etn on lipid and glucose metabolism and liver/gut health in high-fat diet (HFD)–fed mice, complemented by in vitro hepatocyte assays.

**Methods:**

Ten-wk-old C57BL/6 mice (20 male, 18 female) were fed ad libitum HFD (45% energy from fat) with [Ethanolamine supplementation (ES-group)] or without (CON-group) Etn (8 g/kg diet) for 10 wk. Outcomes included body/liver weight, glucose tolerance test (GTT) results, plasma phosphatidylcholine (PC)/cholesteryl ester (CE)/triacylglycerol (TG) concentrations, hepatic TG/PC/PE concentrations, hepatic endoplasmic reticulum (ER)-stress, and inflammation markers, jejunal morphology/barrier/inflammation genes, and fecal microbiota (α/β diversity). HuH7 cells received 20 μM or 5 mM Etn to assess TG/PC/PE synthesis. Statistics: repeated-measures analysis of variance (ANOVA) (GTT), t-test or Wilcoxon (other endpoints), permutational multivariate analysis of variance (PERMANOVA) (β diversity); α=0.05.

**Results:**

ES increased hepatic TG in females by 230% compared with CON (*P* = 0.001), and trended higher in males (*P* = 0.054); hepatic PC and PE masses were unchanged. In ES males, GTT area under the curve decreased by 22.6% (*P* = 0.037), and plasma PC, CE, and TG were reduced by: PC −16.6%, CE −24.5%, TG −25.9%, respectively (all *P* < 0.05). ES males showed higher hepatic *Tnf* and *Cd68* and increased C/EBP homologous protein (CHOP) (all *P* < 0.05). In vitro, Etn did not alter hepatocellular TG, PC, or PE synthesis (all *P* > 0.05). Female ES mice exhibited altered fecal β-diversity (PERMANOVA *P* = 0.006) with early jejunal inflammatory signals (*Tnf* ↑; *P* = 0.055).

**Conclusions:**

Dietary Etn modifies hepatic lipid storage and gut microbiota in a sex-dependent manner and improves glucose tolerance in males, whereas in vitro data indicate no direct effect on hepatocyte lipid synthesis**.**

## Introduction

Metabolic dysfunction-associated steatotic liver disease (MASLD) is a condition characterized by extensive alterations in hepatic lipid metabolism and is often associated with obesity and metabolic syndrome [1]. Among the alterations elicited by MASLD is the overwhelming evidence that hepatic phospholipid composition and metabolism is altered in patients. Studies have found that patients with MASLD have a significantly lower abundance of hepatic phosphatidylcholine (PC) compared with phosphatidylethanolamine (PE) than healthy control patients [2,3]. In the liver, PC and PE make up 40%–50% and 15%–25% of total phospholipids, respectively [4], and are primarily synthesized de novo by the Kennedy pathway [5]. Dietary choline is a metabolic precursor for PC and is recognized as an essential nutrient found in eggs, meat, fish, and soy products, among other foods [6]. In addition to being a substrate for the synthesis of the primary constituent of cell membranes, choline is also required for neurotransmitter synthesis and one-carbon metabolism [7]. Without adequate choline intake, fatty liver is often found in patients due to reduced hepatic PC, impairing the secretion of triacylglycerol (TG) from the liver [8,9]. Previously, our laboratory has shown that when hepatic PC synthesis is reduced by knocking out phosphatidylethanolamine N-methyl transferase (PEMT), the enzyme that converts PE to PC, mice are protected from HFD-induced obesity; however, alterations in the PC/PE ratio lead to impaired VLDL secretion and the development of liver steatosis [9]. In a separate study, we demonstrated that a diet supplemented with high concentrations of choline can prevent the development of fatty liver in PEMT^-/-^ mice [10]. Decreasing PC/PE has also been associated with packing defects on the surface of lipid droplets, which alter the binding of proteins involved in lipolysis and lipid droplet turnover [11], possibly contributing to liver steatosis.

Ethanolamine (Etn), a precursor of PE, is not considered an essential nutrient, and dietary intake in the human population has not been well studied, despite the critical role it plays in phospholipid metabolism. Etn, similar to choline, is present to some degree in most foods, especially animal-based products, which are rich in phospholipids. Notably, data from the milk composition database show that Etn is found in milk at a similar concentration to choline [12]. Etn has been detected in foods, such as black currants, mung beans, lemongrass, and daikon radish, although the concentration has not been quantified [13]. As a naturally occurring and widely distributed organic compound, Etn, as well as its derivatives, play an important role in many important biological processes. As previously mentioned, Etn is critical for the biosynthesis of not only PE but also PC, as PEMT is active in the liver [5], underlining its importance in maintaining liver health. Despite the likelihood that Etn is abundant in the diet, there are currently limited studies surrounding dietary Etn, and it is unclear if Etn supplementation (ES) may decrease the PC/PE ratio and induce or exacerbate MASLD. One recent study by Mishra et al. (2023) [14] found higher accumulation of Etn in the guts of obese mice compared with their lean counterparts, which led the authors to investigate the impacts of short-term ES. They found that in lean mice, 7-d oral gavage treatment with 1 g Etn/kg body weight was sufficient to impair insulin sensitivity and worsen blood glucose recovery following a meal tolerance test [14]. Additionally, mice given Etn displayed decreased expression of the tight junction protein zona-occludens-1 (*Zo1*) in the small intestine, which induced gut permeability and inflammation [14].

To our knowledge, there are currently no studies investigating the impact of long-term dietary intake of Etn. The aim of our study was to examine hepatic and gut health in mice fed a high-fat diet (HFD), with or without ES.

## Methods

### Animals

All procedures were approved by the University of Alberta’s Institutional Animal Care Committee (AUP 175) in accordance with the guidelines of the Canadian Council on Animal Care. Male and female C57BL/6NCrl (*n* = 40) mice were purchased from Charles River Laboratories (Saint Constant, QC). Two male mice were killed early in the trial due to health issues unrelated to the treatment and excluded from further analysis. Mice were exposed to a 12-h light/dark cycle and given free access to food and drinking water. Mice were deprived of food overnight (16 h) prior to tissue collection. Feces were collected from all animals during fasting. Tissues were collected, snap frozen in liquid nitrogen, and stored at −80°C for future analysis. Tissues for histology were fixed in formalin and subject to hematoxylin and eosin (H&E) staining.

### ES

**FIGURE 1:**
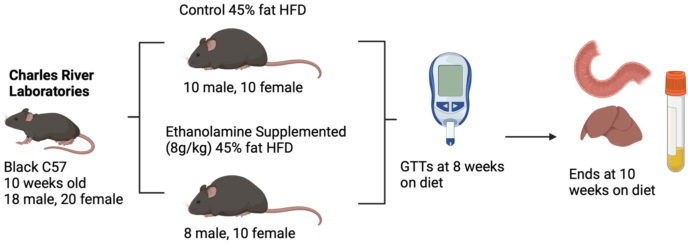
Schematic representation of the dietary intervention and sampling timeline. Mice were assigned to either a high-fat diet control group (10 M, 10 F) or an HFD supplemented with ethanolamine (8 g/kg; 8 M, 10 F). Glucose tolerance tests were conducted after 8 wk on diet, and tissues were collected at week 10. GTT, glucose tolerance test; HFD, high-fat diet.

All mice were fed a HFD containing 45% fat, 35% carbohydrate, 20% protein (#12451 Research Diets) beginning 10 wk after birth and were on diet for 10 wk. ES mice (10F and 8M) received 8 g Etn (Sigma Aldrich #398136) per kg of diet, and control (CON) mice (10F and 10M) did not receive supplementation (Figure 1). Based on mean daily food intake of a 25 g mouse (3–5g) [15], this concentration provided mice with a similar daily Etn dose as the study performed by Mishra et al. [14], and the duration represents chronic Etn exposure that will allow for the observation of semichronic adaptations (gene expression, lipid metabolism).

### In vivo metabolic tests

Glucose tolerance tests (GTTs) were performed at 18 wk of age (8 wk on diet). Mice were deprived of food for 16 h, after which they received glucose (2 g/kg body weight) via intraperitoneal injection. Blood glucose concentrations were measured with glucometer at baseline and at specific time points over 120 min.

### Analysis of total lipid classes

Liver tissue was homogenized in buffer (in mM: 100 Tris·HCl, 150 sodium chloride, 1 EDTA, 1 dithiotheritol (DTT), pH 7.4) containing a protease and phosphatase inhibitor cocktail. Protein concentration of homogenates was quantified using bicinchoninic acid assay kit. Liver homogenates were extracted in the presence of dipalmitoyl-phosphatidyldimethylethanolamine and of batyl alcohol internal standards, using a modification of the method of Folch and Lees [16]. High-performance liquid chromatography (HPLC) was carried out on an Agilent 1100 instrument equipped with quaternary pump and Alltech ELSD 2000 Evaporative Light-Scattering Detector, using a modified version of the method of Abreu et al. (2017) [17]. Lipids were separated using a quaternary solvent system on a 4.6 × 50 mm, 2.7 μm Agilent Poroshell 120 hydrophilic interaction liquid chromatography column (Agilent).

### Immunoblotting

Homogenates were analyzed on SDS-PAGE and transferred to 0.45 μm polyvinylidene fluoride (PVDF) membrane, then probed with primary antibodies against: GAPDH (Ambion #4300, 1:1000; 37kDa), ApoB (Millipore #AB742 1:7500; ∼500kDa for ApoB100, ∼243kDa for ApoB48), eIF2α (Cell Signalling #9722, 1:1000; 38kDa), IRE1α (abcam #ab48187, 1:1000; 110kDa), BiP (Cell Signalling #3183, 1:1000; 78kDa), CHOP (Cell Signalling #2895, 1:1000; 30kDa), and anti-α-tubulin (Sigma #T9026, 1:5000; 55kDa). BiP and CHOP were normalized to GAPDH, and eIF2α and IRE1α were normalized to α-tubulin. Membranes were detected using ECL (WBLUF0500, Millipore) and imaged on Chemi-Doc MP imager (Bio-Rad Laboratories).

### Evaluation of tissue morphology and damage

H&E-stained liver and jejunum tissues were photographed under a light microscope (Zen, AxioCamMR3, Zeiss). Liver tissues were assessed blindly by an independent pathologist for scoring hepatic steatosis, lobular inflammation, portal inflammation, and hepatocellular ballooning using the Brunt criteria [18].

### Plasma blood chemistry

Plasma alanine aminotransferase (ALT) activity was measured using a commercially available kit (Abcam #ab105134). Plasma ketone body concentrations were quantified using a commercially available kit (Abcam #ab272541).

### Gene expression analysis

Total RNA was isolated from liver tissue and jejunal luminal scrapings using TRIzol reagent (Invitrogen #15596018). Total RNA was treated with DNase I (Invitrogen #18068-015) to degrade contaminating genomic DNA, then reverse-transcribed using an oligo(dT)12–18 primer, and Superscript II reverse transcriptase (Invitrogen #18418-012 and 18064014) according to the manufacturer’s instructions. Real-time quantitative PCR was analyzed with Power SYBR Green PCR Master Mix (Thermo Fisher #4367659) in a Step One Plus qPCR system. The data were analyzed with StepOne Software v2.2.2 (Applied Biosystems) by the standard curves method, and mRNA concentrations in liver were normalized to cyclophilin mRNA; mRNA concentrations in jejunum were normalized to ribosomal protein lateral stalk subunit P0 (*Rplpo*) (Supplementary Table 1).

### In vitro hepatocellular ES assessment

To get a more accurate physiological description of the impact of dietary Etn on hepatocytes, we used Huh7 cells stably transfected with mouse PEMT, as Huh7 cells do not express it under normal conditions. Cells were plated at 1 × 10^6^ cells/60 mm dish and grown overnight, then treated with Dulbecco's Modified Eagle Medium (DMEM) containing low (20 μM) or high (5 mM) Etn (Sigma #141-43-5), 0.15 mM oleic acid/bovine serum albumin (BSA), and incubated with 1 μCi/dish ^14^C-oleic acid for 4 h. Cells were harvested after 4 h of pulse treatment. Media on dishes for chase analysis was replaced with media not containing ^14^C-oleic acid. Cells were collected after an 8, 16, and 24 h chase period. Lipid extraction and separation by thin-layer chromatography were carried out as previously described [19]. Radioactivity was measured on TLC plates using AR-2000 TLC Scanner (Eckert & Ziegler). To measure mass of TG, cells were treated for 4 h with DMEM containing low (20 μM) or high (5 mM) Etn and 0.15 mM oleic acid/BSA. Lipids were extracted from cell homogenates as previously described [19], and the lipid layer was dried down under nitrogen gas. Samples were reconstituted in isopropanol, and TG concentration was measured using a commercially available kit (Sekisui Diagnostics #236-60).

### Fecal DNA extraction and 16S rRNA sequencing/analysis

Extraction of DNA from feces was performed using QIAmp DNA Stool Mini Kit (Qiagen #51504) following manufacturer’s instructions with the addition of a bead-beating step. Briefly, ∼100 mg feces were mixed with Inhibitex buffer and 2.0 mm garnet beads (BioSpec Products # 11079103) and lysed twice on a FasPrep-24 homogenizer (MP Biomedicals) at 6.0 m/s for 30 secs. DNA concentration and purity were measured by Nanodrop 2000 (Thermo Scientific) and by Quant-iT PicoGreen dsDNA Assay Kit (Thermo Scientific). The 16S rRNA amplicon sequencing library was prepared following the Illumina 16S Metagenomic Sequencing Library Preparation Protocol targeting the V3-V4 region of the 16S rRNA gene (primers forward 5′ TCG TCG GCA GCG TCA GAT GTG TAT AAG AGA CAG CCT ACG GGN GGC WGC AG and reverse: 5′ GTC TCG TGG GCT CGG AGA TGT GTA TAA GAG ACA GGA CTA CHV GGG TAT CTA ATC C). The final library was diluted to 4 nM, and 2 × 300 bp pair-end metagenome sequencing was performed on an Illumina MiSeq Platform (Illumina Inc.).

Raw sequencing data were processed using Quantitative Insight into Microbial Ecology 2 (QIIME2, v2024.10) [20] and DADA2 [21] as previously described by Marcolla et al., 2019 [22]. Forward and reverse reads were trimmed at 270 and 220 base pairs, respectively, and reads with >6 expected errors were removed. Sequences were aligned, and phylogenetic trees were built using MAFFT [23] and FastTree [24] methods. Taxonomy was assigned using the QIIME 2 feature-classifier plugin [25] with a Naïve Bayes classifier [26] trained on the SILVA 138 database [27]. Amplicon sequence variants (ASVs) were clustered at 99% identity.

Downstream analyses were performed using packages phyloseq (v1.40.0) [28], microbiome (v1.18.0) [29], and qiime2R (v0.99.6) [30] in R (v1.4.1717) [31]. All ASVs identified as Mitochondria, Chloroplast, Archaea, or unassigned were removed, and remaining reads were rarefied to an even sampling depth (females: 16,734; males: 11,381). Alpha-diversity was assessed using phylogenetic diversity, Chao1, Shannon, and Simpson indices. Beta-diversity was evaluated using Bray-Curtis distances and principal coordinates analysis.

Differential abundance analysis was performed using DESeq2 (v1.36.0) [32] at both ASV and taxonomic levels. Taxonomic-level analysis was done by merging ASVs with identical taxonomy strings using the tax_glom function (phyloseq). Packages ggplot2 (v3.4.0) [33] and ggpubr (v0.5.0) [34] were used to generate figures. Analysis of predicted functional potential of the microbiota was performed using phylogenetic investigation of communities by reconstruction of unobserved states (PICRUSt2, v.2.1.4-b) [35] based on Enzyme Commission (EC) numbers and MetaCyc pathways database. Differences in MetaCyc pathways were evaluated using ANOVA-like differential expression (ALDEx) analysis (ALDEx2 package v.1.30.0) [36].

### Statistical analysis

Data are expressed as mean +/- SD (N = 5–10 for each measurement). Repeated measures ANOVA was used to compare blood glucose curves during GTTs, and Student’s t-tests were used for other analyses. Analysis was done using GraphPad Prism 10 software. Alpha-diversity indexes were analyzed using Student’s t-test or the Wilcoxon rank sum test, depending on the normality of data distribution. PERMANOVA was used to evaluate differences in beta-diversity between groups using the vegan package (v. 2.5–7) in R Studio [37,38]. A *P* value < 0.05 was taken as significant.

## Results

### ES improves glucose tolerance in HFD-fed male mice, but worsens hepatic lipid accumulation, inflammation, and oxidative stress

**FIGURE 2:**
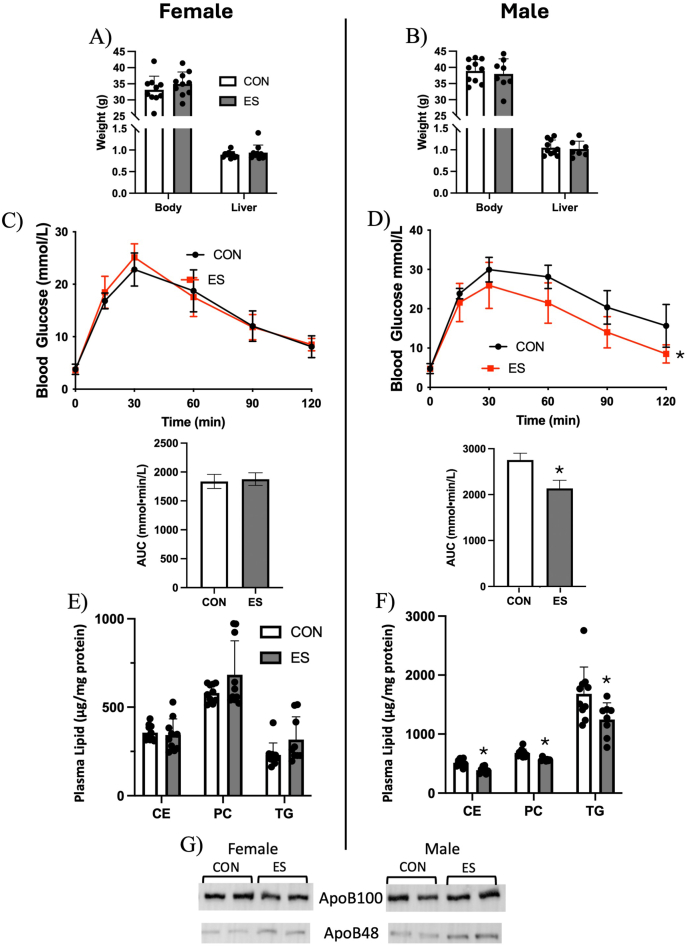
Effects of Etn on weight, glucose tolerance, and plasma lipids in female and male mice. Body and liver weight of female (A) and male (B) CON and ES mice. Glucose tolerance test (GTT) of female (C) and male (D) CON and ES mice. Mass of plasma TG, PC, and CE in (E) female and (F) male CON and ES mice. (G) Plasma ApoB100 and ApoB48 expression in CON vs ES mice. *N* = 5 per group for GTT and *N* = 8–10 for other analyses. ∗Significant difference and *P* < 0.05 was taken as significant. AUC, area under the curve; CE, cholesteryl esters; CON, control; ES, ethanolamine supplemented; PC, phosphatidylcholine; TG, triglycerides; GTT, glucose tolerance test.

**FIGURE 3:**
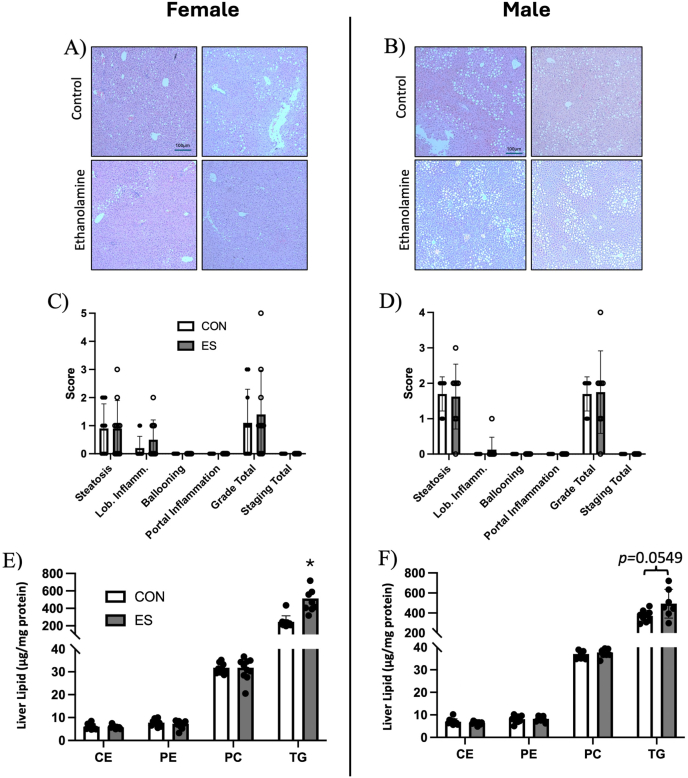
Hepatic lipids and histologic examination of CON and ES mice. H&E-stained liver tissues of female (A) and male (B) mice. Histologic grading of common characteristics of MASLD, including steatosis and lobular inflammation in female (C) and male (D) mice. Mass of CE, PE, PC, and TG in the liver of CON and ES female (E) and male (F) mice. *N* = 5–10 per group. ∗Significant difference and *P* < 0.05 was taken as significant. H&E, hematoxylin and eosin; CE, cholesteryl esters; CON, control; ES, ethanolamine supplemented; PC, phosphatidylcholine; PE, phosphatidylethanolamine; TG, triglycerides.

**FIGURE 4:**
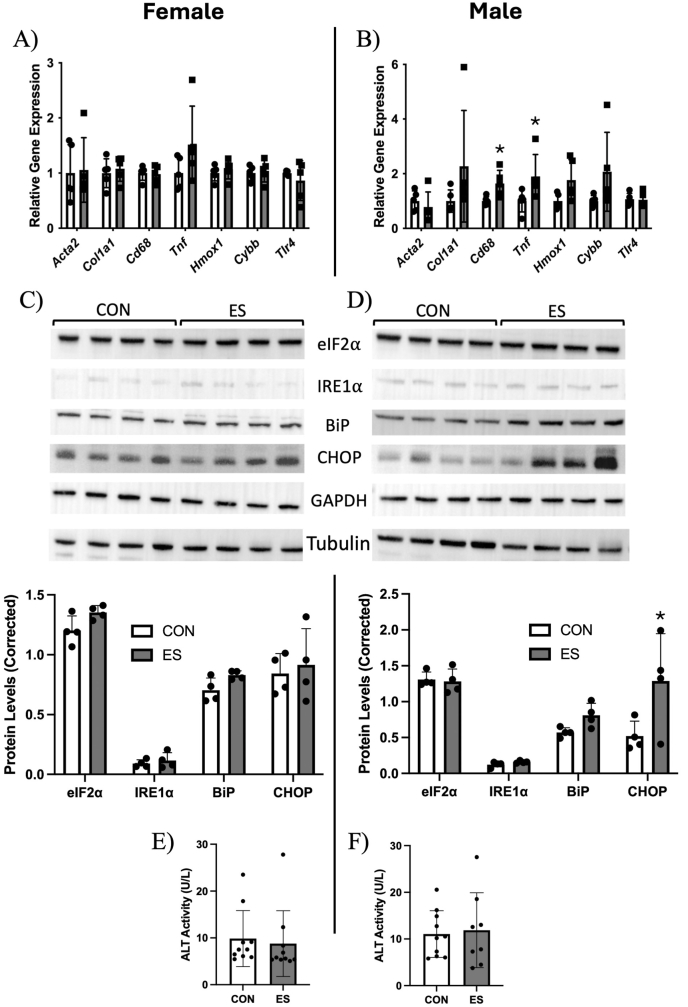
Markers of liver health, fibrosis, inflammation, and oxidative stress in CON and ES mice. Gene markers for fibrosis, inflammation, and oxidative stress were measured in female (A) and male (B) control and ES mice. Hepatic protein expression of markers for oxidative stress in female (C) and male (D) CON and ES mice. Female (E) and male (F) plasma ALT concentrations of CON and ES mice. *N* = 5 per group mRNA concentrations. ∗Significant difference and *P* < 0.05 was taken as significant. *Acta2*, Actin alpha 2, smooth muscle; BiP, binding immunoglobulin protein; *Cd68*, Cluster of Differentiation 68; CHOP, C/EBP Homologous Protein; *Col1a1*, Collagen, Type 1, Alpha 1; CON, control; *Cybb*, cytochrome b-245 beta chain; eIF2a, Eukaryotic Initiation Factor 2A; ES, ethanolamine supplemented; GAPDH, Glyceraldehyde-3-phosphate dehydrogenase; *Hmox1*, heme oxygenase 1; IRE1α, inositol-requiring enzyme 1 alpha; *Tlr4*, toll-like receptor 4; *Tnf*, tumor necrosis factor.

At the end of the feeding trial, there were no differences observed in body weight or liver weight between CON and ES mice of either sex (Figure 2A, B). Glucose tolerance between CON and ES female mice was not significantly different (Figure 2C); however, there was a significant difference between glucose tolerance curves between the CON and ES male mice, with ES mice displaying improved glucose tolerance (Figure 2D). Plasma lipids were unaltered in female ES mice (Figure 2E), but plasma PC, CE, and TG were reduced in male ES mice compared with CON (Figure 2F). To explain reduced plasma lipids in male mice, we measured plasma ApoB expression. We found no difference in ApoB100 expression in females or males, and ApoB48 expression was increased in the plasma of male ES mice (Figure 2G). Because glucose metabolism and plasma lipids were altered in male ES mice, we next used histologic grading of the liver to assess the characteristics of MASLD. Visualization of liver tissue by H&E staining under a light microscope showed similar accumulation of lipids between treatment groups in both males and females (Figure 3 A, B). To confirm these observations, tissue was subject to examination by an independent pathologist, and it was determined that ES did not significantly alter histologic grading of steatosis or lobular inflammation, and there was no evidence of portal inflammation or hepatocellular ballooning in males and females (Figure 3C, D). Although we did not see any obvious differences in hepatic lipid accumulation, we measured total mass of hepatic lipids. Results show that PE, PC, and CE mass in the liver was not impacted by ES; however, hepatic TG was increased in female ES mice (Figure 3E) and trended higher in male ES mice (*P* = 0.0549) (Figure 3F). To further examine the impact of ES on liver health, we measured hepatic mRNA expression of genes related to fibrosis, inflammation, and oxidative stress. We found no significant differences due to treatment in the expression of *Acta2*, *Col1A1, Cd68, Tnf, Hmox1*, or *Tlr4* in the female mice (Figure 4A). In male mice, genes related to fibrosis (*Acta2/Col1A1*) and oxidative stress (*Hmox1/Cybb*) were unaltered; however, we found a significant elevation in the expression of inflammatory markers *Tnf* and *Cd68*, but not *Tlr4,* in ES male mice (Figure 4B). To further evaluate liver damage, protein expression of markers of hepatic oxidative stress in female and male CON and ES mice was assessed. Expression concentrations of eIF2α and IRE1α did not differ significantly between groups. BiP and CHOP expression concentrations remained unchanged in female mice across both dietary conditions (Figure 4C). In contrast, male mice supplemented with Etn exhibited a significant increase in CHOP protein concentrations compared to male control mice (Figure 4D), indicating enhanced endoplasmic reticulum (ER) stress in response to ES. As a marker of general liver damage, we measured plasma ALT activity and found no significant difference in activity between control and ES females or males (Figure 4E, F). All values were within the normal range and did not indicate liver damage.

### ES alters genes related to hepatic lipid and glucose metabolism in male mice fed HFD

**FIGURE 5:**
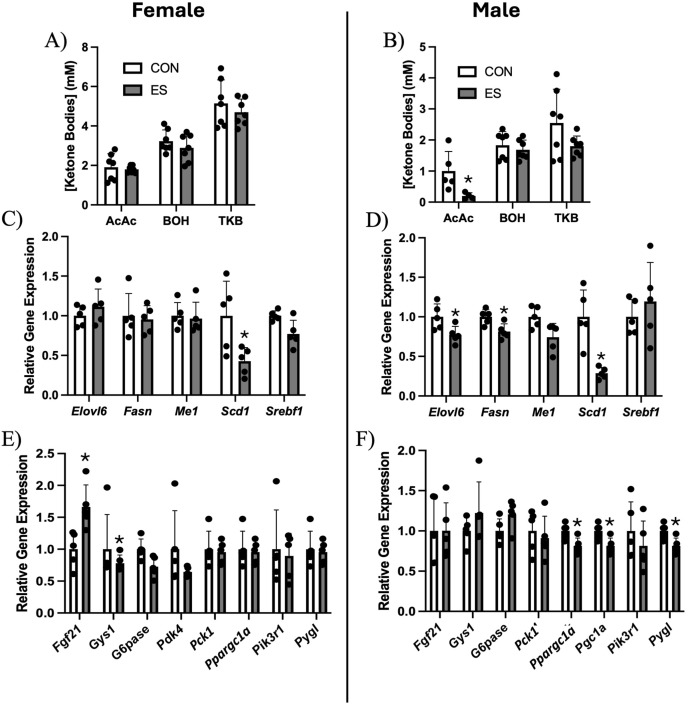
Markers of altered hepatic lipid and glucose metabolism in CON and ES mice. Plasma concentration of AcAc, BOH, and total ketone bodies in females (A) and males (B). Gene expression of genes involved in hepatic lipid synthesis in female (C) and male (D) mice. Expression of genes related to gluconeogenesis, glycogenesis, glycogenolysis, and ketogenesis in female (E) and male (F) mice. *N* = 5–7 per group. ∗significant difference and *P* < 0.05 was taken as significant. AcAc, acetoacetate; BOH, beta-hydroxybutyrate; CON, control; *Elovl6*, elongation of very long-chain fatty acids protein 6; ES, ethanolamine supplemented; *Fasn*, fatty acid synthase; *Fgf21;* fibroblast growth factor 21; *Gys1*, glycogen synthase 1; *G6pase*, glucose-6-phosphatase; *Me1*; malic enzyme 1; *Pck1*, phosphoenolpyruvate carboxykinase 1; *Pdk4*, pyruvate dehydrogenase kinase; *Pik3r1*, Phosphoinositide-3-kinase Regulatory Subunit 1; *Ppargc1a*, peroxisome proliferator-activated receptor gamma coactivator 1-alpha; *Pygl*, glycogen phosphorylase L; *Scd1*, Stearoyl-CoA desaturase 1; *Srebf1*; sterol regulatory element binding transcription factor 1; TKB, total ketone bodies.

To further investigate the impact of ES on hepatic lipid metabolism and energy homeostasis, we measured the concentration of ketone bodies present in the plasma. In female mice, concentrations of acetoacetate (AcAc), β-hydroxybutyrate (BOH), and total ketone bodies (TKBs) were not different in ES mice (Figure 5A). In male mice, plasma [AcAc] was significantly reduced in ES mice; however, [BOH] and [TKB] were unaltered (Figure 5B). To investigate the molecular mechanisms underlying the observed differences in hepatic lipid accumulation and glucose tolerance, we analyzed the expression of genes involved in hepatic lipid metabolism and glucose regulation. Interestingly, *Scd1,* a gene linked to increased hepatic TG accumulation, was decreased in both female and male ES mice (Figure 5C, D). In male mice, *Elovl6* and *Fasn* were also decreased by ES (Figure 5D). When investigating the impact of Etn on genes related to glucose metabolism, we observed an increase in *Fgf21* and a decrease in *Gys1* in female ES mice (Figure 5E); however, these differences were not found in males. In male ES mice, genes related to gluconeogenesis (*Pck1)*, fatty acid oxidation (*Ppargc1a),* and glycogen breakdown (*Pygl*) were reduced (Figure 5F).

### ES does not have a direct impact on hepatocytes in vitro

**FIGURE 6:**
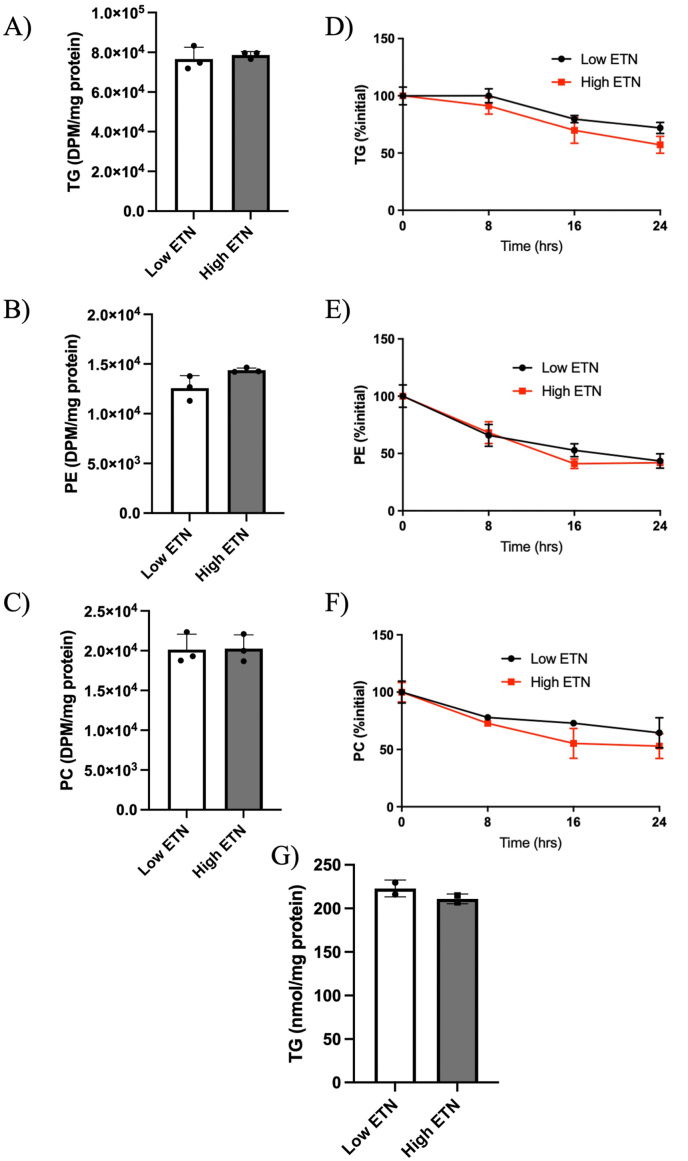
Assessment of the effects of Etn on lipid metabolism in Huh7 cells. ^14^C-labeled TG (A), PE (B), and PC (C) in Huh7 cells treated with 20 μM or 5 mM Etn after a 4 h incubation with ^14^C-oleic acid. Turnover of labeled TG (D), PE (E), and PC (F) expressed as % initial over a 24 h chase period following 4 h pulse. Total cellular mass of TG in Huh7 cells treated with 20 μM or 5 mM Etn (G). *N* = 3–6 per group. ∗Significant difference and *P* < 0.05 was taken as significant. ETN, ethanolamine; PC, phosphatidylcholine; PE, phosphatidylethanolamine; TG, triglycerides.

To assess if Etn treatment in hepatocytes impacts hepatic lipid metabolism, we treated Huh7 cells with physiological (20 μM) and excess (5 mM) concentrations of Etn. We found no differences in the synthesis of TG, PE, or PC after 4 h when ^14^C-oleic acid incorporation was measured (Figure 6A–C). There were also no differences in the turnover of these lipids over a 24 h chase period (Figure 6D–F). As an added measure of lipid accumulation in the cells, we measured cellular TG concentration and found no differences between the low and high Etn treatments (Figure 6G).

### Beta-diversity of fecal microbiota is altered by ES in female HFD-fed mice, but not males

**FIGURE 7:**
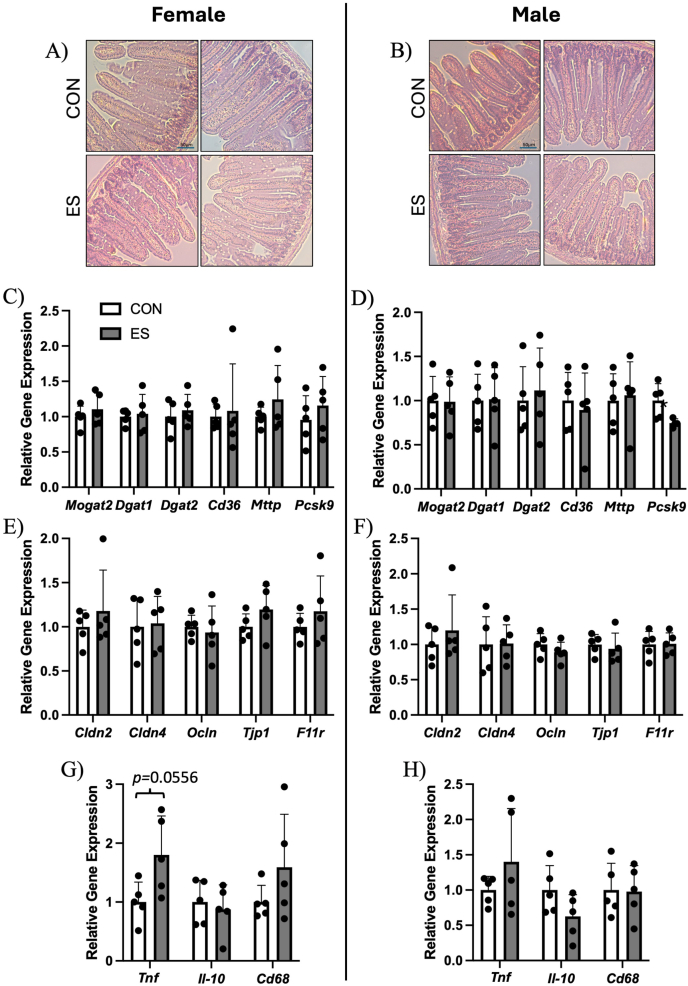
Histologic analysis and expression of genes related to gut function and inflammation in the jejunum of female and male CON and ES mice. H&E-stained jejunum tissue of female (A) and male (B) CON and ES mice. Jejunal expression of genes involved in lipid synthesis and metabolism in female (C) and male (D) CON and ES mice. Expression of genes related to gut barrier function in the jejunum of female (E) and male (F) CON and ES mice. mRNA expression of markers of inflammation in the jejunum of female (G) and male (H) CON and ES mice. *N* = 5 per group. ∗Significant difference and *P* < 0.05 was taken as significant. H&E, hematoxylin and eosin; CON, control; *Cd36*, cluster of differentiation 36*; Cd68*, cluster of differentiation 68*; Cldn2,* Claudin 2*; Cldn4,* Claudin 4*; Dgat1*, diacylglycerol O-acyltransferase 1*; Dgat2*, diacylglycerol O-acyltransferase 2*;* ES, ethanolamine supplemented; *F11r*, F11 receptor*; Il-10*, interleukin-10*; Mogat*, monoacylglycerol O-acyltransferase 1; *Mttp*, microsomal triglyceride transfer protein*; Ocln*, occludin*; Pcsk9*, proprotein convertase subtilisin/kexin type 9; *Tjp1*, tight junction protein 1.

**FIGURE 8:**
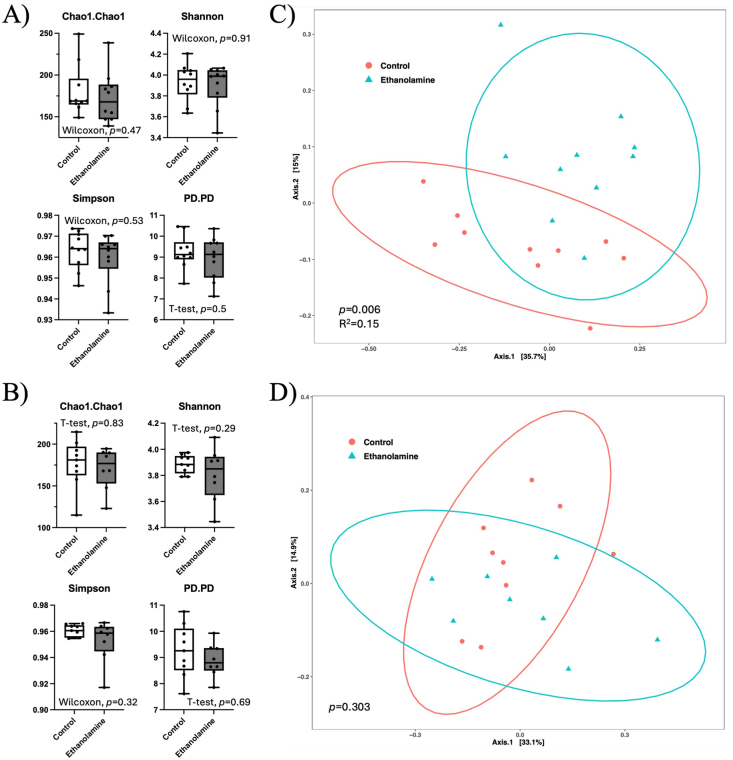
Measures of alpha- and beta-diversity in microbiota of CON and ES mice. (A, B) Alpha-diversity indices and (C, D) principal coordinates analysis were generated based on Bray-Curtis dissimilarity in female (A, C) and male (B, D) CON and ES mice. *N* = 8–10 per group. CON, control; ES, ethanolamine supplemented.

**FIGURE 9:**
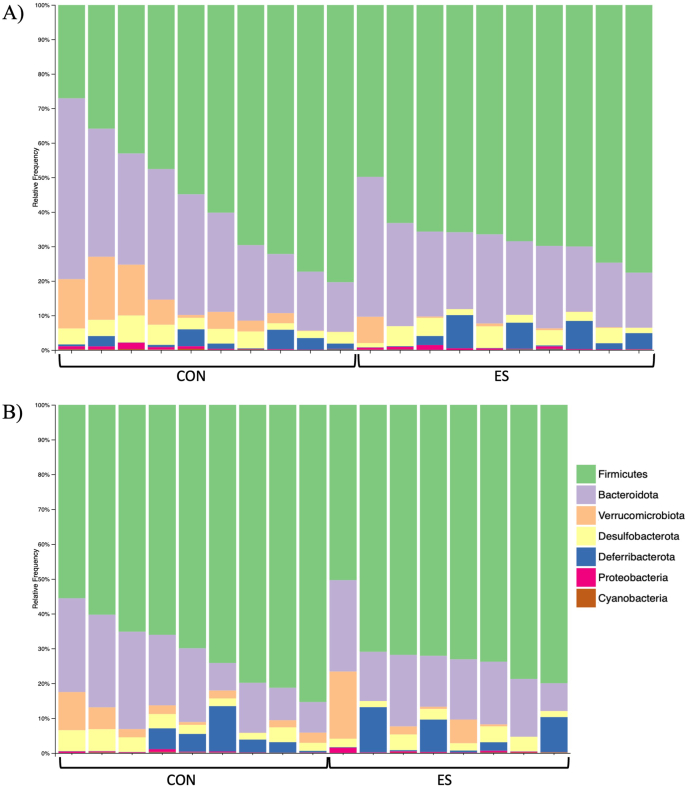
Fecal microbiome composition in CON and ES mice. Taxa bar plot of relative abundance of phyla in fecal samples from female (A) and male (B) control and ES mice. *N* = 8–10 per group. CON, control; ES, ethanolamine supplemented.

To investigate if the impact of ES on lipid metabolism and glucose regulation was related to intestinal health/function, we performed a histologic evaluation of the morphology and architecture of the jejunum. In both female and male mice, we observed no qualitative differences in villi length or crypt depth between control and ES mice (Figure 7A, B). Because we observed alterations in liver lipids in our mice, as well as plasma lipids in males, we measured expression of genes related to intestinal lipid metabolism. In females, we found no differences between CON and ES mice (Figure 7C); most lipid-related genes were unaltered in male ES mice, except *Pcsk9*, which was reduced (Figure 7D). To assess gut barrier function, we measured gene expression of tight junction proteins in the jejunum of our mice. We found no differences between dietary groups in either sex (Figure 7E, F). Notably, we did not see any difference in expression of *Tjp1*, a tight junction protein supposedly reduced by Etn treatment [14]. Expression of genes related to intestinal inflammation was not significantly impacted by the ES diet in either sex; however, increased *Tnf* expression in females neared significance (*P =* 0.0556) (Figure 7G, H). Because alterations in the microbiome are known to impact metabolic health, we investigated the impact of the ES diet on the fecal microbiota composition in our HFD fed mice. We found no differences in measures of alpha-diversity, including phylogenetic diversity in either sex (Figure 8A, B). Assessment of beta-diversity by measure of Bray-Curtis dissimilarity showed that microbiota composition was significantly different between control and ES female mice (Figure 8C); however, no differences were observed between control and ES male mice (Figure 8D). A taxa bar plot shows the relative abundance of microbial phyla in which no differences were seen at the phylum level in either females or males (Figure 9A, B) (Supplementary Tables 2, 3). PICRUSt2 analysis indicated 4 EC to be significantly enriched in the Etn treatment. The enriched enzymes are involved in starch, sucrose, fructose, mannose, and glycerolipid metabolism pathways (Supplementary Table 4).

## Discussion

Previous studies have shown that Etn accumulation in the gut induces endotoxemia, leaky gut, and glucose intolerance, and that oral Etn treatment leads to a decline in glucose sensitivity [14]. The primary purpose of our study was to determine if dietary Etn impacts hepatic lipid metabolism in HFD in mice. It was previously reported that short-term oral Etn gavage negatively impacted both gut and whole-body metabolism; however, the long-term impact of dietary Etn has not been explored. We supplemented mice with Etn in the diet for 10 wk, which is a sufficient duration to describe any impact of chronic exposure, as described in other nutritional intervention studies [39,40]. Based on concentrations used in previous literature where mice have been supplemented with choline [41,42], and on the dose of Etn gavaged by Mishra et al. [14], we supplemented an HFD with 8 g of Etn per kg of diet. We found that although the ES diet did not impact body weight or liver weight in our mice, there was an improvement in glucose tolerance in male mice (Figure 2D), contrary to previous reports [14]. In general, it has been documented that males are more prone to diet-induced insulin resistance and may be more sensitive to alterations in diet composition [43]; in this study, it appears that CON female mice did not display HFD-induced glucose intolerance to the same extent as males, which may contribute to the lack of improvement in glucose sensitivity in ES females. In addition to improved glucose tolerance in ES males, we observed a reduction in plasma CE, TG, and PC in males (Figure 2F). The link between improved glucose sensitivity and reduced plasma lipids is well recognized, and may suggest a decrease in circulating VLDL, similar to findings in animals fed a choline-deficient diet [44,45]; however, we saw no difference in plasma ApoB100 and an increase in ApoB48 in male ES mice (Figure 2G). This suggests alterations in chylomicron clearance by the liver or altered chylomicron assembly in the intestine, resulting in smaller, lipid-poor particles [46,47].

Previous studies by our group investigated the impact of phospholipid metabolism on metabolic health and found that *Pemt*^-/-^ mice are protected from HFD-induced insulin resistance, but display increased hepatic lipid accumulation due to impaired VLDL secretion resulting from a decrease in the PC/PE ratio in the liver [9,[48], [49], [50]]. It was hypothesized that increased availability of Etn would increase PE synthesis by increasing flux through the Kennedy pathway; however, there were no differences in hepatic PC or PE mass or the hepatic PC/PE. As a protective measure, hepatocytes will regulate enzymatic activity to maintain a healthy PC/PE ratio, so it is possible that PEMT activity is upregulated in ES mice to balance the influx of PE synthesized [51]. Although we observed no differences in the histologic grading of MASLD in our CON and ES animals, we did find an increase in hepatic lipid accumulation in ES mice (Figure 3), opposite to what would be expected when animals are displaying improved glucose sensitivity [52]. Increased TG accumulation with no differences in histologic signs of MASLD suggests that dietary Etn may promote hepatic lipid storage; however, the length of this study was not enough for the development of fibrosis. Upon further examination, we found signs of inflammation (*Tnf, Cd68*) and ER stress (CHOP) in male ES mice (Figure 4B, D), indicating that Etn promotes the unfolded protein response, similar to mice fed a methionine-choline-deficient diet [53]. Interestingly, despite an increase in hepatic TG accumulation, we observed a decrease in genes related to de novo lipogenesis (Figure 5C, D). This suggests that hepatic TG accumulation in ES mice is not a result of increased hepatic lipogenesis. Previous work from our laboratory shows that altering PE metabolism may increase TG accumulation in hepatocytes, despite decreased expression of de novo lipogenesis (DNL)-related genes, possibly due to a reduction in fatty acid oxidation [19]. *Scd1* was significantly reduced in both ES females and males. The protein encoded by this gene, stearoyl-CoA desaturase 1 (SCD1), is responsible for desaturating saturated fatty acids (SFA) such as stearate and palmitate, which may have lipotoxic effects on the cell; therefore, reductions in *Scd1* expression may be associated with ER stress and activation of the UPR [54]. Altered abundance of SFAs compared with unsaturated fatty acids may alter the fatty acid species in phospholipids such as PE, further attenuating ER stress due to alterations in membrane fluidity [55]. Reductions in *Scd1* expression have also been shown to improve insulin sensitivity by decreasing G6Pase and PEPCK expression, whereas increasing hepatic TG accumulation [56]. In ES male mice, we also observed a decrease in genes related to gluconeogenesis (*Pck1, Ppargc1a*) and glycogenolysis (*Pygl*) that was not seen in female ES mice, outlining a possible mechanism for the observed improvement in glucose tolerance that was specific to males. The lack of differences in the incorporation of oleic acid into TG, PC, and PE in hepatoma-derived (Huh7) cells upon Etn treatment suggests that ES does not directly impact hepatic lipid metabolism (Figure 6).

Previous studies suggest that Etn accumulates in the gut and can act to increase gut permeability, resulting in inflammation and impaired glucose sensitivity, which could be reversed by reintroduction of Etn metabolizing microbiota [14]. Gut permeability and alterations in the gut microbiota have been linked to insulin resistance pathogenesis, with the proposed mechanism being the release of LPS into the circulation from the gut, leading to endotoxemia and inflammation [57]. Etn is abundant in the gut, due to the breakdown of PE from diet by microbiota [58] and is catabolized by bacteria in the gut that carry the Etn utilization (*eut)* operon, which breaks the molecule down into acetaldehyde and ammonium to provide the bacteria a source of carbon and nitrogen [58]. The microbiota of obese mice have been shown to display a decreased abundance of Etn metabolizing bacteria in the gut compared to lean controls, leading to Etn accumulation that induces gut permeability [14]. Contrary to previous findings using oral Etn administration, we see no alterations in the expression of tight junction proteins encoding genes such as *Tjp1* (Figure 7E, F); however, we do see a trend for increased jejunal inflammation, specifically in female ES mice (Figure 7G).

Sex differences in the hormonal-gut axis may contribute to variability in gut microbiome composition and function. Sex hormones, particularly estrogen and testosterone, influence gut permeability, immune responses, and microbial diversity. These interactions can affect metabolic and neuroendocrine processes differently in males and females, potentially shaping disease susceptibility and treatment responses. Understanding these sex-specific mechanisms is, therefore, critical for developing personalized therapeutic strategies targeting the gut microbiome. We observed a significant alteration in the microbiota composition of female ES mice (Figure 8C) along with an increased relative abundance of *Erysipelatoclostridiaceae* taxa compared with CON female mice (Supplementary Table 3). Interestingly, a species in this genus, *Erysipelatoclostridium ramosum,* has been linked to the exacerbation of obesity in HFD-fed mice [59]. Other taxa that were enriched in ES females compared with CON females in the current study have previously been linked to positive health effects. Notably, we observed an increased abundance of *Oscillospiraceae* species, which produce short-chain fatty acids and are associated with reduced risk of type 2 diabetes and obesity [60]. Alterations in gut microbiota may partially explain why female ES mice seem to be protected from hepatic oxidative stress; however, future studies focusing on microbial functional capacity are needed to explain differences in metabolic phenotypes between CON and ES male mice.

Although this study provides new insight into the effects of dietary Etn on metabolic health, several limitations should be considered. The 10-wk feeding period represents moderate chronic exposure and may not reflect long-term outcomes, such as fibrosis or advanced steatohepatitis. Although sex-specific responses were observed, the study was not powered to identify the mechanisms underlying these differences, which may involve hormonal regulation, microbial metabolism of Etn, or phospholipid synthesis. The in vitro experiments used immortalized hepatoma cells, which do not fully capture primary hepatocyte function or systemic influences present in vivo. Additionally, 16S rRNA sequencing provided information on microbiota composition but not function; metagenomic or metabolomic analyses would better link microbial shifts with host metabolism. Finally, the Etn dose (8 g/kg diet) may exceed typical dietary exposure, and dose–response studies are needed to determine physiological relevance.

Overall, although dietary Etn may not directly impact PE metabolism as previously hypothesized, sex-specific metabolic changes were observed on ES. Previous studies have administered Etn by oral gavage for >7 d and observed impaired glucose tolerance and increased gut permeability. Our study shows that long-term feeding with ES does not provide the same results and highlights the importance of sex consideration in metabolic studies. Future studies will be required to determine the mechanism by which dietary Etn alters the metabolic profile in females and males.

## Author contributions

The authors’ responsibilities were as follows – RLJ: designed research and had primary responsibility for final content; CMH, AV, KL, RN, AT, YF: conducted research; CMH, AV, CSM, RLJ: analyzed data and wrote paper; KL, RN, YF, CSM, RDC, BPW, RLJ: reviewed final manuscript and provided necessary corrections; all authors: read and approved the final manuscript.

## Data availability statement

Data described in the manuscript can be made available upon request pending approval.

## Funding

This project was funded by the Canadian Institutes of Health Research (CIHR).

HPLC experiments were performed at the University of Alberta Faculty of Medicine & Dentistry Lipidomics Core, RRID:SCR_019176, which receives financial support from the Faculty of Medicine & Dentistry, Canada Foundation for Innovation (CFI), and the Natural Sciences and Engineering Research Council of Canada (NSERC) awards to contributing investigators.

## Conflict of interests

The authors report no conflicts of interest.
