# Supplementary material for: Dietary Ethanolamine Increases Hepatic Lipid Accumulation in Mice Fed a High-Fat Diet
Source: J Nutr. 2026 Jan 8;156(3):101348. doi: 10.1016/j.tjnut.2025.101348 (PMC13014508; doi:10.1016/j.tjnut.2025.101348)
Supplement: Multimedia component 1 [file mmc1.docx]

**Supplementary Table. 1 List of Mouse Primers Used for qPCR Gene Analysis.**

**Supplementary Table 2. Different Abundance of Fecal Microbial 16s rRNA in Female Mice**

**Supplementary Table 3. Different Abundance of Fecal Microbial 16s rRNA in Male Mice**

**Supplementary Table 4. PICRUSt2 Analysis Results of Enzymes Enriched in ES Mice**
